# Prediction of Carcass Traits of Hair Sheep Lambs Using Body Measurements

**DOI:** 10.3390/ani10081276

**Published:** 2020-07-27

**Authors:** Emmanuel Bautista-Díaz, Jesús Alberto Mezo-Solis, José Herrera-Camacho, Aldenamar Cruz-Hernández, Armando Gomez-Vazquez, Luis Orlindo Tedeschi, Héctor Aarón Lee-Rangel, Einar Vargas-Bello-Pérez, Alfonso Juventino Chay-Canul

**Affiliations:** 1 División Académica de Ciencias Agropecuarias, Universidad Juárez Autónoma de Tabasco, Carretera Villahermosa-Teapa Km 25, Villahermosa 86280, Tabasco, Mexico; emmanuel_09_27@hotmail.com (E.B.-D.); jesusmezo.solis@gmail.com (J.A.M.-S.); ingaldecruz@gmail.com (A.C.-H.); dragv2@hotmail.com (A.G.-V.); 2 Instituto de Investigaciones Agropecuarias y Forestales, Universidad Michoacana de San Nicolás de Hidalgo, Carretera Morelia-Zinapécuaro Km 9.5, El Trébol, Tarímbaro 58893, Michoacán, Mexico; josheca@gmail.com; 3 Department of Animal Science, Texas A&M University, College Station, TX 77843-2471, USA; luis.tedeschi@tamu.edu; 4 Facultad de Agronomía y Veterinaria, Universidad Autónoma de San Luis Potosí, San Luis Potosí 78000, S.L.P., Mexico; hector.lee@uaslp.mx; 5 Department of Veterinary and Animal Sciences, Faculty of Health and Medical Sciences, University of Copenhagen, Grønnegårdsvej 3, DK-1870 Frederiksberg C, Denmark

**Keywords:** body measurements, carcass traits, prediction, linear traits, modelling

## Abstract

**Simple Summary:**

Some authors have reported that the use of body measurements (BMs) could be a useful tool for predicting carcass characteristics in sheep. Hair sheep breeds have been adopted for lamb production in the tropical regions of Latin America. Among these, Pelibuey and Katahdin breeds and their crosses have shown great reproductive capacity and adaptation, contributing to improving the productive efficiency of flocks in tropical production systems. However, few studies have been carried out on this breeds to define its BMs correctly, and little work has been found using BMs to predict the carcass characteristics in different physiological stages.

**Abstract:**

The present study was designed to evaluate the relationship between the body measurements (BMs) and carcass characteristics of hair sheep lambs. Twenty hours before slaughter, the shrunk body weight (SBW) and BMs were recorded. The BMs involved were height at withers (HW), rib depth (RD), body diagonal length (BDL), body length (BL), pelvic girdle length (PGL), rump depth (RuD), rump height (RH), pin-bone width (PBW), hook-bone width (HBW), abdomen width (AW), girth (GC), and abdomen circumference (AC). After slaughter, the carcasses were weighed and chilled for 24 h at 1 °C, and then were split by the dorsal midline. The left-half was dissected into total soft tissues (muscle + fat; TST) and bone (BON), which were weighed separately. The weights of viscera and organs (VIS), internal fat (IF), and offals (OFF—skin, head, feet, tail, and blood) were also recorded. The equations obtained for predicting SBW, HCW, and CCW had an *r*^2^ ranging from 0.89 to 0.99, and those for predicting the TST and BON had an *r*^2^ ranging from 0.74 to 0.91, demonstrating satisfactory accuracy. Our results indicated that use of BMs could accurately and precisely be used as a useful tool for predicting carcass characteristics of hair sheep lambs.

## 1. Introduction

Alternative or indirect methods for the determination of the carcass and body composition of livestock have recently been studied [1,2]. Among the most common indirect variables to determine the carcass and body composition of sheep are body weight (BW) and body condition score (BCS). On the other hand, direct methods involve the separation and dissection of an animal’s body and the determination of its physical and chemical components [1,2]. However, these latter methods are extremely laborious, time-consuming, and costly because of the large number of personnel and laboratory analyses required in addition to being wasteful, as half of the carcass is subsequently discarded [1,2,3,4].

Indirect methods include the prediction of carcass and body composition based on parameters easily obtained [1] through ultrasound [4,5,6], computed tomography, dual x-ray absorptiometry (DEXA), digital image analysis or body measurements [6,7,8,9]. Some of these methods, such as computerised tomography, magnetic resonance, and DEXA are limited to developed countries due to the cost of acquiring the necessary equipment and the need for specialised, professionally trained personnel; also, these methods can be time-consuming [3,9,10,11]. Ultimately, the selected method for predicting carcass traits and body composition should be based on several factors, including the cost, ease of adoption and prediction accuracy regardless of the sex, age, or diet of animals [1].

In vivo body measurements (BMs) may be of greater interest under commercial production conditions because they enable the assessment of carcass traits or the prediction of commercial cuts with few or no additional costs to producers [8,10,12]. Due to their low cost and practicality, BMs have long been used as an indicator of animal type and potential of production [13,14] or as a predictor of BW, body mass index, and carcass traits [15,16,17]. However, the use of BMs to estimate growth and carcass and body composition in hair sheep is limited [8]. Hence, the present study was aimed to evaluate the relationship between body measurements (BMs) and carcass traits in hair sheep lambs.

## 2. Materials and Methods

### 2.1. Location and Animals Management

The animals were treated in accordance with the guidelines and regulations for animal experimentation of the División Académica de Ciencias Agropecuarias, Universidad Juárez Autónoma de Tabasco (ID project PFI: UJAT-DACA-2015-IA-02). The experiment was carried out at the El Rodeo commercial farm (17°84″ N, 92°81″ W; 10 m a.s.l.) located at km 14 of the Villahermosa-Jalapa highway, Tabasco, Mexico. The study was performed on 66 hair lambs, including 39 Pelibuey and 27 Katahdin sheep. Of these, 36 were male and 30 were female; 30 were single lambings and 36 were double lambings. Ewes and lambs were housed together in individual pens of 1.5 × 2 m. They were held for 56 days prior with access to food and water. The offspring did not have direct access to the ewes’ feeders. Lambs were slaughtered at 56 days of age.

### 2.2. Body Measurements

**Figure 1:**
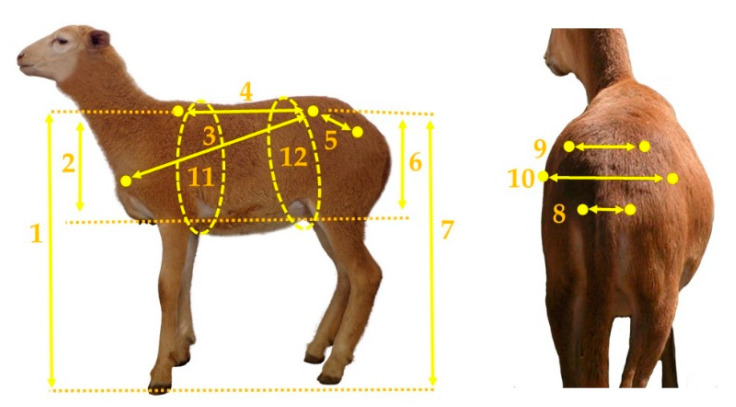
Body measurements taken in hair sheep lambs. (**1**) height at withers, (**2**) rib depth, (**3**) body diagonal length, (**4**) body length, (**5**) pelvic girdle length, (**6**) rump depth, (**7**) rump height, (**8**) pin bone width, (**9**) hook bone width, (**10**) abdomen width, (**11**) girth circumference, and (**12**) abdomen circumference. The lamb used as a reference was 56 days of age and 14.2 kg of live weight.

For each lamb, the following body measurements (BMs) were recorded 24 h prior to slaughter. The BMs were taken as described previously by Bautista-Diaz et al. [8]: (1) height at withers (HW), (2) rib depth (RD), (3) body diagonal length (BDL), (4) body length (BL), (5) pelvic girdle length (PGL), (6) rump depth (RuD), (7) rump height (RH), (8) pin bone width (PBW), (9) hook bone width (HBW), (10) abdomen width (AW), (11) girth circumference (GC), and (12) abdomen circumference (AC) (Figure 1). Flexible fiberglass tape (Truper^®^) and large 65-cm calipers (Haglof^®^) were used to perform the measurements. BMs were expressed in cm, and the measurements taken were previously related to carcass composition [8].

### 2.3. Slaughter of Animals

Lambs were fasted for 20 hours before slaughtering in order to record shrunk body weight (SBW). Lambs were slaughtered according to the Mexican Official Standard NOM-033-ZOO-1995 for the humane slaughter of animals. After slaughter, the carcasses were weighed (hot carcass weight; HCW). Then, they were split by the dorsal midline into two halves and kept at 1 °C. Then, after 24 h, carcasses were reweighed (cold carcass weight; CCW), and the left halves were dissected into total soft tissues (muscle + fat; TSTs) and bone tissues (BONs), which were each weighed separately. The weights of the tissues dissected from the left halves of the carcass were doubled to reflect the total carcass weight. The viscera and organs (VIS: liver, heart, kidneys, lungs, rumen and empty intestines, gall bladder, and spleen) were removed and weighed. Internal fat (IF) was grouped as pelvic fat (around kidneys and the pelvic region) or as fat around the gastrointestinal tract (omental and mesenteric). The gastrointestinal tract (GIT) was weighed full and empty. The empty BW (EBW) was calculated as the slaughter body weight minus the GIT contents. Finally, the weight of the waste parts (skin, head, feet, tail, and blood; OFF) was recorded.

### 2.4. Statistical Analyses 

A descriptive statistical analysis was performed using the PROC MEANS procedure in SAS, North Carolina, USA [18]. Correlation coefficients of Pearson’s among variables were estimated using the PROC CORR procedure in SAS SAS, North Carolina, USA [18]. Regressions were developed using the PROC REG procedure in SAS, North Carolina, USA [18]. The STEPWISE option and Mallow’s Cp were used in the SELECTION statement to select the variables included in the model. The goodness of fit of the developed models was evaluated by the determination coefficients (*r*^2^) and root mean squared error (RMSE).

Based on the recommendations of Tedeschi [19], several additional statistics were used to assess the predictability of the equations, including the coefficients of determination (*r*^2^), mean squared error (MSE), standard deviation (SD), mean squared error of prediction (MSEP) and root of the MSEP (RMSEP), which account for the distance between predicted and true values. The regressions were evaluated according to the null hypothesis (H_0_) that states that b_0_ is equal to zero and b_1_ is equal to one and the alternative hypothesis (H_A_) that is not H_0_. A non-rejection of the null hypothesis means that the model accurately explained the variation that occurred in the dataset. The precision was assessed by the evaluation of the *r*^2^ of the linear regression of *Y* (i.e., observed) on *X* (i.e., predicted) as described by Fonseca et al. [20] and Morales-Martinez et al. [4]. The mean bias (MB), as described by Cochran and Cox [21], was used as a representation of the average inaccuracy of the model. The modelling efficiency factor (MEF), which represents the proportion of variation explained by the line *Y* = *X*, was used as an indicator of goodness of fit [22,23]. The coefficient of model determination (CD) was used to assess variance in the predicted data. The bias correction factor (Cb), a component of the concordance correlation coefficient (CCC) [24], was used as an indicator of deviation from the identity line, and the CCCs were also used as a reproducibility index to account for accuracy and precision. High accuracy and precision were assumed when the coefficients were >0.80, and low accuracy and precision were assumed when the coefficients were <0.50. These calculations were performed in the Model Evaluation System proposed by Tedeschi [19].

## 3. Results and Discussion

The descriptive statistics of the BMs and carcass characteristics are reported in Table 1. The SBW ranged from 6.08 to 16.85 kg (coefficient of variation (CV) of 23.9%). With respect to the carcass weights (HCW and CCW), the HCW ranged from 2.85 kg to 8.56 kg, with a CV around 26% for both parameters. The weights of carcass tissues presented high variability (Table 1): The CVs of the TST and BON weights were 30.5% and 18.4%, respectively, and even greater variation was observed in the IF weight, which had a CV > 64. Internal fat is considered to be the most variable body component. Several authors have stated that body fat varies due to factors such as breed, sex, age and state of maturity [8,17,25,26]. Finally, low to moderate variation was observed in the BMs (6.44–19.216%).

The PBW and RD were not correlated with IF and VIS, respectively (*P* > 0.05). Nonetheless, the other MB have an *r* that varied from 0.24 to 0.84 (*P* < 0.05) (Table 2). The regression equations developed to predict the carcass characteristics are presented in Table 3: For both HCW and CCW, three equations were obtained, with an *r*^2^ ranging from 0.89 to 0.99 (Table 2); in these models, the AC, RD, and PGL were included (*P* < 0.05). For the prediction of TST, BON, VIS, and IF, the equations had an *r*^2^ ranging from 0.47 to 0.98; in these models, the GC, RD, HBW, PGL, AC, and RuD were included. For the prediction of OFF weight, only one the equation was fitted, with an *r*^2^ of 0.94; in this case, only SBW was included as a predictor. Notably, SBW accounted for a high proportion of the variation in the carcass traits evaluated in the present study (47% to 99%; Table 3). Previously, the importance of SBW as a predictor was reported for cattle [6]. Notably, the SBW accounted for 47% to 99% of the variation in the evaluated carcass traits (Table 2).

The inclusion of BMs such as GC, RD, HBW, PGL, AC, and RuD to the prediction equations improved the *r*^2^ or reduced the RMSE (Table 2). According to previous research, BMs alone can explain between 38% and 93% of the variation in the carcass traits of ewes [8]. In hair sheep, few studies have used BMs to predict the carcass traits of animals in different physiological states. However, of these studies, it was reported that the HW of animals was a good predictor of carcass weight, true carcass yield, CCW and viscera weight [8,27]. Nonetheless, in the present study, the HW was not included in any model. Similarly, Hernández-Espinoza et al. [26] informed that the TST weight had a negative relationship with BL. However, in the current study, the BL had a positive association with the TST weight (*r* = 0.65, *P* < 0.001). Nevertheless, it was not included as a predictor in any equation used to predict TST weight (Table 2), nor were any of the BMs related with body length (BL or BDL) included in the models for predicting carcass traits (Table 3). This is in agreement with the previous reports of Hernández-Espinoza et al. [27] and Bautista-Díaz et al. [8]. On the other hand, in adult Pelibuey ewes, Bautista-Díaz et al. (2017) found that GC and AC showed a good correlation with HCW and CCW in agreement with the results found for hair lambs in the present study.

Concerning other breeds of sheep, Shehata [28] reported that the GC accounted for around 72% of the variation in the carcass weights (HCW and CCW) in Barki lambs. Also, this latter author found that GC was a good predictor of the primary commercial cuts, accounting for 45% to 67% of the variation. In buffalo calves, Rashad et al. [29] found that the GC had a high correlation with all carcass characteristics except bone weight; but was the best predictor of boneless meat and carcass fat weights (*r*^2^ = 0.90, 0.78). In Pelibuey ewes, Bautista-Díaz et al. [8] reported that BM can be used to predict the carcass tissue composition (in weights). Nonetheless, these authors do not recommend that their models be used for animals of the opposite sex or with distinct physiological conditions.

**Figure 2:**
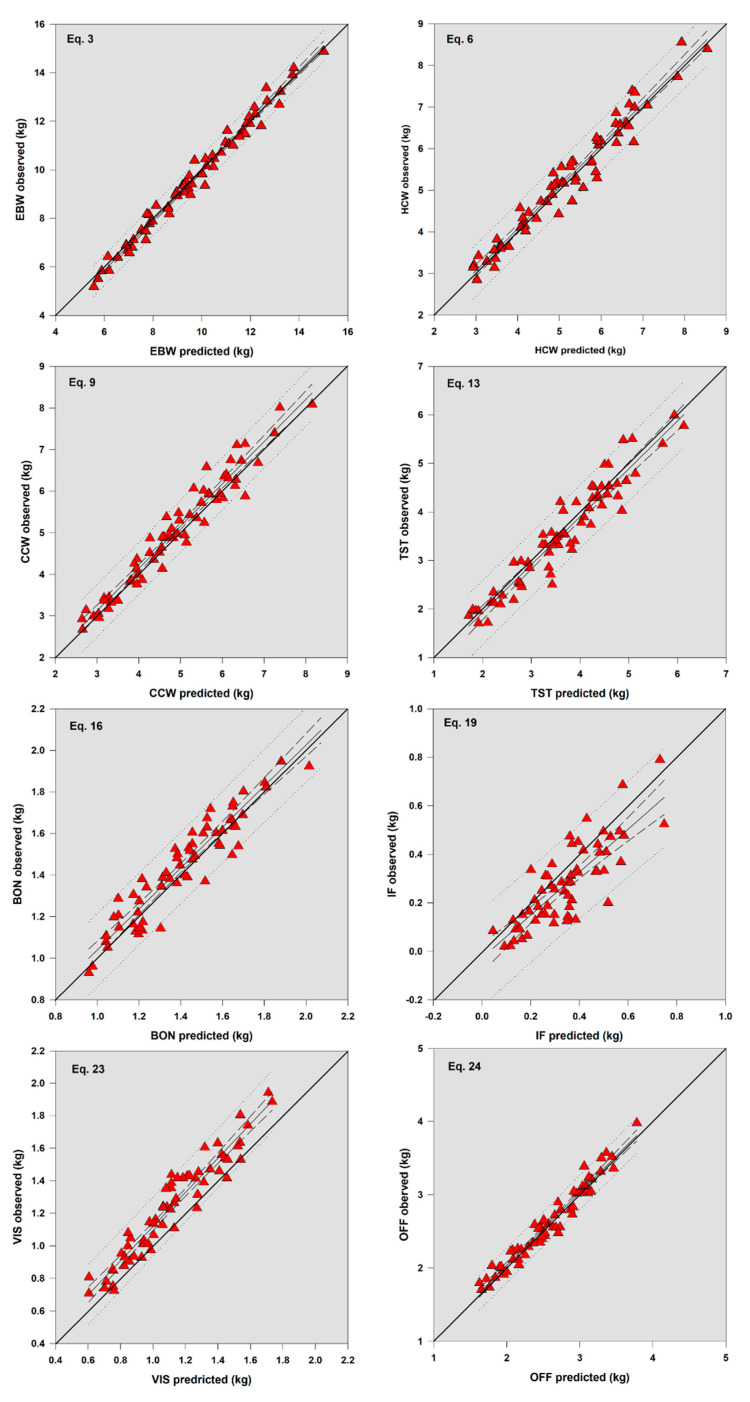
Relationship between observed and predicted values of carcass traits in suckling hair lambs.

Finally, in regard to the evaluation of the equations (Table 4, Figure 2), all equations presented high precision (*r*^2^ > 0.90), except for Equations (16) and (19) (Table 4; Figure 2). In addition, all equations presented high accuracy (Cb > 0.90; Table 3) and had a good reproducibility index and good concordance with the observed data (CCC = 0.99, 0.97, 0.93, 0.95, 0.91, 0.76, 0.86, and 0.97 for Equations (3)–(24), respectively). According to the MEF, all equations except for Equation (19) presented a moderate to the high efficiency of prediction (from 0.71 to 0.98), indicating a moderate to high concordance between the predicted and actual values. It is important to note the MEF has been reported as the best measure of concordance between observed and predicted values. Meanwhile, the CDs of the equations ranged from 0.98 to 1.16, indicating high variability in the predicted data (Table 4; Figure 2). In this case, a model with a perfect fit would have a CD value of one, and values closer to one indicate improvement in the predictions of a model. The present values indicate an underestimation of the carcass traits, with a variation of about 16% [19], considering that a CD > 1 indicates underprediction and a CD < 1 indicates overprediction. The main component of the MSEP for Equations (3), (6), (9), (13), (16), (19) and (24) was random error (91.1, 92.2, 76.6, 90.8, 80.1, 74.8, and 87.0, respectively), although for Equation (23), a high proportion of the MSEP was attributed to mean bias (65.10%; Table 4, Figure 2).

## 4. Conclusions

The equations for predicting shrunk body weight (SBW), hot carcass weight (HCW), and cold carcass weight (CCW) using BMs had an *r*^2^ ranging from 0.89 to 0.99, and those for predicting the weights of the total soft tissues (TST) and bone tissues (BON) had an *r*^2^ ranging from 0.74 to 0.91. According to the evaluation parameters, these equations have satisfactory accuracy. Hence, the use of BMs could accurately and precisely be used as a useful and practical tool for predicting carcass characteristics of hair sheep lambs.

## Figures and Tables

**Table 1:** Descriptive analyses of the body measurements of live animals (n = 66 suckling lambs).

Variable	Description	Mean ± SD	Maximum	Minimum	CV (%)
*Body measurements*					
SBW	Shrunk body weight (kg)	10.78 ± 2.58	16.85	6.08	23.93
EBW	Empty body weight (kg)	9.64 ± 2.33	14.88	5.18	24.18
HW	Height at withers (cm)	48.47 ± 3.46	56.00	34.00	7.14
RD	Rib depth (cm)	17.96 ± 2.68	26.00	14.00	14.92
BDL	Body diagonal length (cm)	36.68 ± 3.13	44.00	29.00	8.53
BL	Body length (cm)	29.29 ± 2.61	35.00	23.00	8.91
PGL	Pelvic girdle length (cm)	13.55 ± 2.14	17.00	10.00	15.79
RuD	Rump depth (cm)	15.20 ± 2.92	23.00	8.00	19.21
RH	Rump height (cm)	48.00 ± 3.09	55.00	41.00	6.44
PBW	Pin bone width (cm)	5.72 ± 1.08	8.00	3.50	18.88
HBW	Hook bone width (cm)	8.84 ± 1.18	12.50	6.60	13.35
AW	Abdomen width (cm)	10.92 ± 1.58	14.00	7.00	14.47
GC	Girth circumference (cm)	51.22 ± 4.63	61.00	34.00	9.04
AC	Abdomen circumference (cm)	51.33 ± 5.63	65.00	40.00	10.97
*Carcass characteristics*					
HCW	Hot carcass weight (kg)	5.28 ± 1.36	8.56	2.85	25.76
CCW	Cold carcass weight (kg)	5.02 ± 1.33	8.09	2.68	26.49
TST	Total soft tissues (muscle + fat), (kg)	3.54 ± 1.08	5.99	1.71	30.51
BON	Bone (kg)	1.46 ± 0.27	2.25	0.93	18.49
IF	Internal fat (kg)	0.28 ± 0.18	0.79	0.02	64.29
VIS	Organs and viscera (kg)	1.26 ± 0.32	2.25	0.71	25.40
OFF	Offals (kg)	2.61 ± 0.54	3.98	1.71	20.69

SD = standard deviation; CV: coefficient of variation.

**Table 2:** Pearson correlations coefficients of between body measurements and carcass traits of suckling hair lambs ^1^.

	EBW	HCW	CCW	TST	BON	IF	VIS	OFF	HW	RD	BL	BDL	PGL	RuD	RH	GC	AC	PBW	HBW	AW
SBW	0.98	0.95	0.92	0.90	0.86	0.68	0.84	0.96	0.68	0.32 **	0.75	0.79	0.49	0.49	0.62	0.87	0.84	0.47	0.67	0.72
EBW		0.96	0.94	0.93	0.85	0.73	0.80	0.95	0.68	0.34 **	0.72	0.77	0.46	0.46	0.61	0.88	0.81	0.45	0.66	0.70
HCW			0.96	0.95	0.84	0.76	0.72	0.93	0.65	0.40	0.70	0.76	0.38 **	0.38 **	0.58	0.84	0.74	0.40	0.67	0.70
CCW				0.98	0.88	0.75	0.69	0.90	0.65	0.36 **	0.67	0.72	0.38 **	0.37 **	0.57	0.81	0.68	0.33 **	0.64	0.65
TST					0.83	0.77	0.64	0.88	0.63	0.34 **	0.65	0.70	0.37 **	0.34 *	0.54	0.81	0.66	0.31 *	0.64	0.65
BON						0.54	0.69	0.85	0.68	0.41	0.63	0.66	0.33 **	0.39 **	0.62	0.70	0.69	0.38 **	0.61	0.61
IF							0.45	0.61	0.45	0.29 *	0.39	0.56	0.24 *	0.27 *	0.30 *	0.63	0.41	0.17 ^ns^	0.53	0.51
VIS								0.82	0.55	0.12 ^ns^	0.72	0.62	0.49	0.58	0.51	0.72	0.83	0.50	0.47 *	0.57
OFF									0.64	0.31 **	0.72	0.76	0.48	0.48 *	0.56	0.83	0.79	0.46 *	0.63	0.69
HW										0.50	0.64	0.67	0.25 *	0.21 ^ns^	0.77	0.66	0.66	0.42	0.59	0.47
RD											0.22 ^ns^	0.37 *	0.039 **	−0.36 **	0.60	0.41	0.34 **	0.19 ^ns^	0.44	0.39
BL												0.60	0.45	0.38 **	0.57	0.69	0.70	0.49	0.53	0.57
BDL													0.31 **	0.25 *	0.59	0.74	0.72	0.41	0.53	0.63
PGL														0.68	0.07 ^ns^	0.40	0.37 **	0.24 *	0.20 ^ns^	0.22 ^ns^
RuD															0.11 ^ns^	0.30 *	0.41	0.39	0.34 **	0.31 **
RH																0.63	0.62	0.46	0.50	0.45
GC																	0.81	0.47	0.63	0.70
AC																		0.55	0.64	0.69
PBW																			0.39 **	0.39 **
HBW																				0.64

^1^ Correlations followed by no superscript indicate *P* < 0.001; **: *P* < 0.01; *: *P* < 0.05; ns: non-significant.

**Table 3:** Regression equations for prediction of carcass traits in suckling hair lambs using body measurements ^1^.

No. Equation	Equation	n	RMSE	*r* ^2^	*P*
*EBW*					
1	EBW (kg) = 0.89 (±0.004 ***) × SBW	66	0.34	0.99	<0.0001
2	EBW (kg) = 1.29 (±0.46 **) + 0.97 (±0.03 ***) × SBW − 0.04 (±0.01 **) × AC	66	0.33	0.98	<0.0001
3	EBW (kg) = 0.91 (±0.02 ***) × SBW + 0.04 (±0.01 ***) × GC − 0.04 (±0.01 ***) × AC	65	0.29	0.99	<0.0001
*HCW*					
4	HCW (kg) = 0.49(±0.004 ***) × SBW	66	0.40	0.99	<0.0001
5	HCW (kg) = 1.78 (±0.52 **) + 0.62 (±0.03 ***) × SBW − 0.06 (±0.01 **) × AC	66	0.37	0.92	<0.0001
6	HCW (kg) = 1.13 (±0.46 *) + 0.61 (±0.03 ***) × SBW + 0.06 (±0.02 ***) × RD − 0.07 (±0.01 ***) × AC	65	0.30	0.95	<0.0001
*CCW*					
7	CCW (kg) = 0.47 (±0.005 ***) × SBW	66	0.50	0.99	<0.0001
8	CCW (kg) = 2.45 (±0.63 ***) + 0.63 (±0.04 ***) × SBW − 0.08 (±0.02 ***) × AC	66	0.45	0.89	<0.0001
9	CCW (kg) = 2.53 (±0.52 ***) + 0.65 (±0.03 ***) × SBW − 0.08 (±0.02 ***) × PGL − 0.07 (±0.01 ***) × AC	64	0.31	0.94	<0.0001
*TST*					
10	TST (kg) = −0.55 (±0.24 *) + 0.38 (±0.02 ***) × SBW	66	0.46	0.82	<0.0001
11	TST (kg) = 1.66 (±0.58 **) + 0.51 (±0.03 ***) × SBW − 0.07 (±0.02 ***) × AC	66	0.41	0.85	<0.0001
12	TST (kg) = 2.16 (±0.58 ***) + 0.54 (±0.04 ***) × SBW − 0.05 (±0.02 **) × RuD − 0.07 (±0.02 ***)AC	66	0.39	0.87	<0.0001
13	TST (kg) = 1.52 (±0.53 **) + 0.53 (±0.03 ***) × SBW − 0.06 (±0.02 ***) × RuD − 0.07 (±0.01 ***) × AC + 0.10 (±0.05 *) × HBW	65	0.33	0.91	<0.0001
*BON*					
14	BON (kg) = 0.47 (±0.07 ***) + 0.09 (±0.01 ***) × SBW	66	0.14	0.74	<0.0001
15	BON (kg) = 0.26 (±0.12 *) + 0.09 (±0.01 ***) × SBW + 0.01 (±0.01 *) × RD	66	0.14	0.76	<0.0001
16	BON (kg) = 0.79 (±0.20 ***) + 0.12 (±0.01 ***) × SBW + 0.02 (±0.01 ***) × RD − 0.02 (±0.01 ***) × GC	64	0.10	0.86	<0.0001
IF					
17	IF (kg) = −0.24 (±0.07 **) + 0.05 (±0.01 ***) × SBW	66	0.14	0.47	<0.0001
18	IF (kg) = 0.37 (±0.17 *) + 0.09 (±0.01 ***) × SBW − 0.02 (±0.01 ***) × AC	66	0.11	0.65	<0.0001
19	IF (kg) = 0.08 (±0.008 ***) × SBW + 0.01 (±0.004 **) × GC − 0.02 (±0.004 ***) × AC	64	0.10	0.90	<0.0001
*VIS*					
20	VIS (kg) = 0.11 (±0.002 ***) × SBW	66	0.17	0.98	<0.0001
21	VIS (kg) = −0.66 (±0.23 **) + 0.06 (±0.02 ***) × SBW + 0.03 (±0.01 ***) × AC	66	0.17	0.76	<0.0001
22	VIS (kg) = −0.89 (±0.22 ***) + 0.05 (±0.01 **) × SBW + 0.02 (±0.01 ***) × RuD + 0.03 (±0.01 ***) × AC	66	0.14	0.79	<0.0001
23	VIS (kg) = −0.53 (±0.15 **) + 0.07 (±0.01 ***) × SBW + 0.02 (±0.004 ***) × RuD + 0.02 (±0.004 ***) × AC − 0.05 (±0.02 **) × HBW	62	0.09	0.90	<0.0001
OFF					
24	OFF (kg) = 0.41 (±0.07 ***) + 0.20 (±0.01 ***) × SBW	64	0.12	0.94	<0.0001

^1^ Values within parentheses are the SEs of the parameter estimates. *: *P* < 0.05; **: *P* < 0.01; ***: *P* < 0.001. Intercepts not different from 0 were removed from the final equations. EBW: empty body weight (kg); HCW: hot carcass weight (kg); CCW: cold carcass weight (kg); TST: total soft tissues (muscle + fat, kg); BON: bone tissues (kg); IF: internal fat (kg); VIS: organs and viscera (kg); OFF: offals (kg); SBW: shrunk body weight (kg); AC: abdomen circumference (cm); GC: girth circumference (cm); RD: rib depth (cm); PGL: pelvic girdle length (cm); RuD: rump depth (cm); HBW: hook bone width (cm); MSE: mean square error; RMSE: root mean square error; *r*^2^: coefficient of determination.

**Table 4:** Mean and descriptive statistics of the accuracy and precision of the equations for predicting carcass traits in suckling hair lambs using body measurements.

Variable ^1^	Equation (3) EBW	Equation (6) HCW	Equation (9) CCW	Equation (13) TST	Equation (16) BON	Equation (19) IF	Equation (23) VIS	Equation (24) OFF
Mean	9.64	5.18	4.88	3.62	1.42	0.34	1.11	2.56
SD	2.24	1.32	1.28	1.05	0.25	0.16	0.28	0.52
Maximum	15.01	8.54	8.15	6.13	2.07	0.75	1.73	3.78
Minimum	5.57	2.94	2.64	1.72	0.96	−0.02	0.61	1.63
*r* ^2^	0.98	0.95	0.94	0.91	0.86	0.66	0.90	0.94
CCC	0.99	0.97	0.93	0.95	0.91	0.76	0.86	0.97
Cb	0.99	0.99	0.98	0.99	0.98	0.94	0.90	0.99
MEF	0.98	0.95	0.93	0.90	0.84	0.56	0.71	0.94
CD	1.07	1.05	1.07	1.05	1.14	1.16	0.98	1.08
Regression analysis								
Intercept (β_0_)								
Estimate	−0.30	0.06	0.07	−0.04	0.02	−0.03	0.08	−0.01
SE	0.16	0.15	0.15	0.14	0.07	0.03	0.05	0.07
*P*-value (β_0_ = 0)	0.07	0.69	0.61	0.75	0.68	0.32	0.09	0.93
Slope (β_1_)								
Estimate	1.02	1.00	1.01	0.98	1.00	0.93	1.03	1.02
SE	0.02	0.03	0.03	0.04	0.05	0.08	0.04	0.03
*P*-value (β_1_ = 1)	0.12	0.91	0.59	0.72	0.85	0.41	0.38	0.54
MSEP source, % MSEP								
Mean bias	2.39	6.36	20.67	8.14	16.01	21.51	65.10	9.68
Systematic bias	6.47	1.46	2.64	1.04	3.80	3.63	2.58	3.31
Random error	91.13	92.61	76.68	90.81	80.18	74.85	32.32	87.00
Root MSEP								
Estimate	0.09	0.09	0.11	0.11	0.01	0.01	0.03	0.02
% of the mean	3.22	5.89	7.00	9.18	7.57	35.19	14.38	5.06

^1^ Obs: observed evaluation data set; CCC: concordance correlation coefficient; Cb: bias correction factor; MEF: modelling efficiency; CD: coefficient of model determination; MSEP: mean square error of the prediction; EBW: empty body weight (kg); HCW: hot carcass weight (kg); CCW: cold carcass weight (kg); TST: total soft tissues (muscle + fat, kg); BON: bone tissues (kg); IF: internal fat (kg); VIS: organs and viscera (kg); OFF: offals (kg).
